# Supplementary material for: Linkage mapping reveals loci that underlie differences in Caenorhabditis elegans growth
Source: G3 (Bethesda). 2022 Aug 12;12(10):jkac207. doi: 10.1093/g3journal/jkac207 (PMC9526057; doi:10.1093/g3journal/jkac207)
Supplement: jkac207_Supplementary_Data [file jkac207_supplementary_data.pdf]

Supplementary material

Linkage mapping reveals loci that underlie differences in *C. elegans* growth

Joy Nyaanga[1,2] and Erik C. Andersen[1*]

1. Department of Molecular Biosciences, Northwestern University, Evanston, IL 60208, USA
2. Interdisciplinary Biological Sciences Program, Northwestern University, Evanston, IL 60208, USA

S1 File. Raw animal growth data.
Raw growth data collected from the COPAS BIOSORT and processed using the *easysorter* R package to compile information from each well. (CSV)
S2 File. Pruned animal growth data.
Processed data from the COPAS BIOSORT following implementation of the *mclust* R package and removal of clusters containing non-animal objects. (CSV)
S3 File. RIAIL phenotype data.
Residual phenotypic values for RIAILs, and parent strains (N2 and CB4856) for each trait (TOF = length and normEXT = width). (TSV)
S4 File. Linkage mapping results.
Linkage mapping results with annotated QTL and confidence intervals. (CSV)
S5 File. CSS/NIL sequence data.
VCF from the whole-genome sequencing for all the CSSs/NILs in this study. (DOCX)
S6 File. CSS/NIL genotype data.
Genotypes for the CSSs/NILs in this study. (CSV)
S7 File. CSS and NIL raw phenotype data.
Raw phenotype data for the CSSs and NILs in this study. (CSV)
S8 File. CSS and NIL pruned phenotype data.
Pruned phenotype data for the CSSs and NILs in this study. (CSV)
S9 File. Statistical significance for mean animal length from CSS/NIL assay.
Pairwise statistical significance for all strains. (CSV)
S10 File. Statistical significance for mean animal width from CSS/NIL assay.
Pairwise statistical significance for all strains. (CSV)
S11 File. List of genes in the chromosome X QTL.
List of all genes in the chromosome X interval, their functional descriptions and GP annotations, and whether they have variation in the CB4856. (CSV)
S1 Text. Reagents used to generate CSSs and NILs. (PDF)

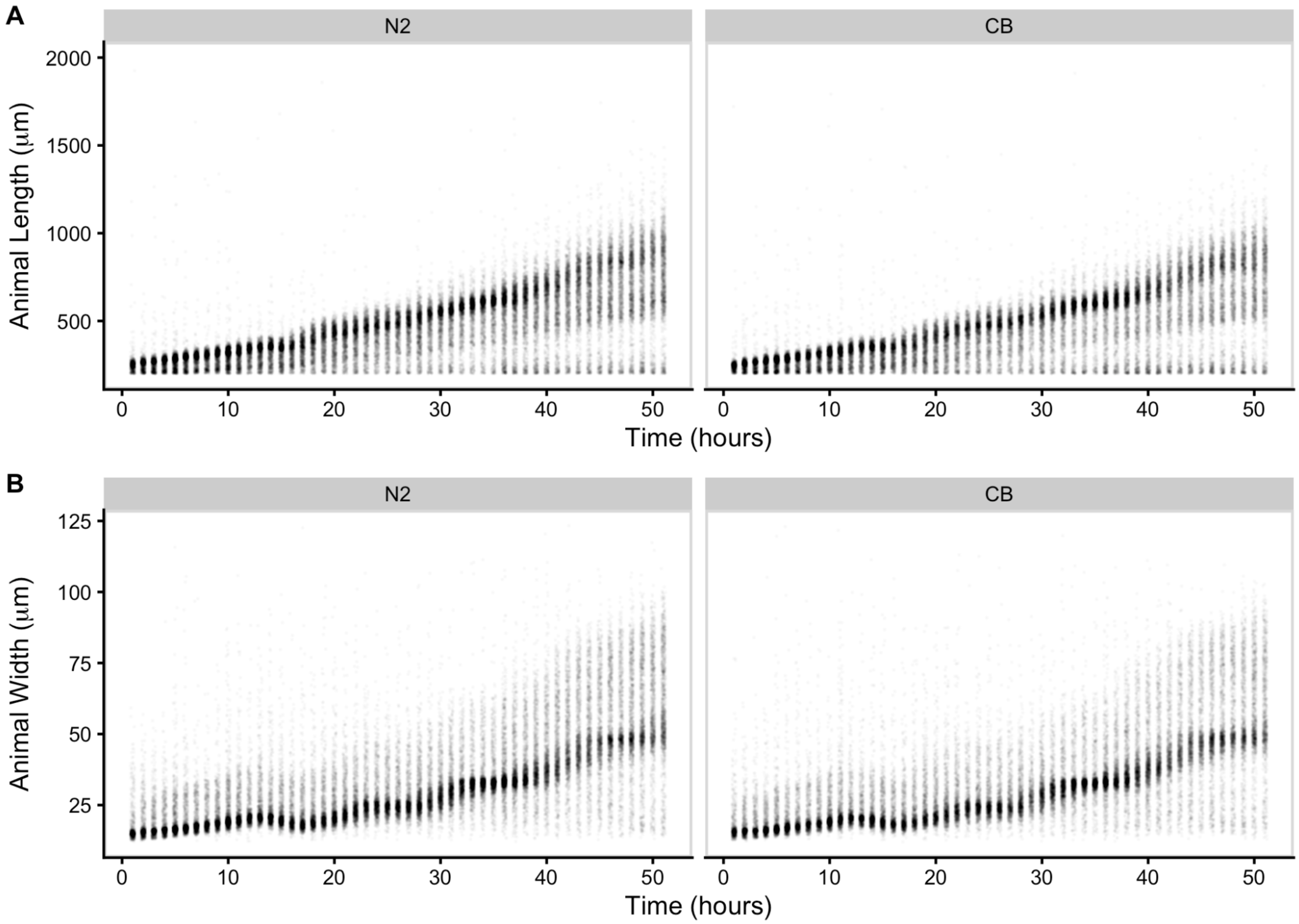

S1 Fig. Raw measurements of animal size.

Raw COPAS BIOSORT of animal length (A) and width (B) for N2 and CB4856 objects are shown here. Each point represents an individual object that was measured.

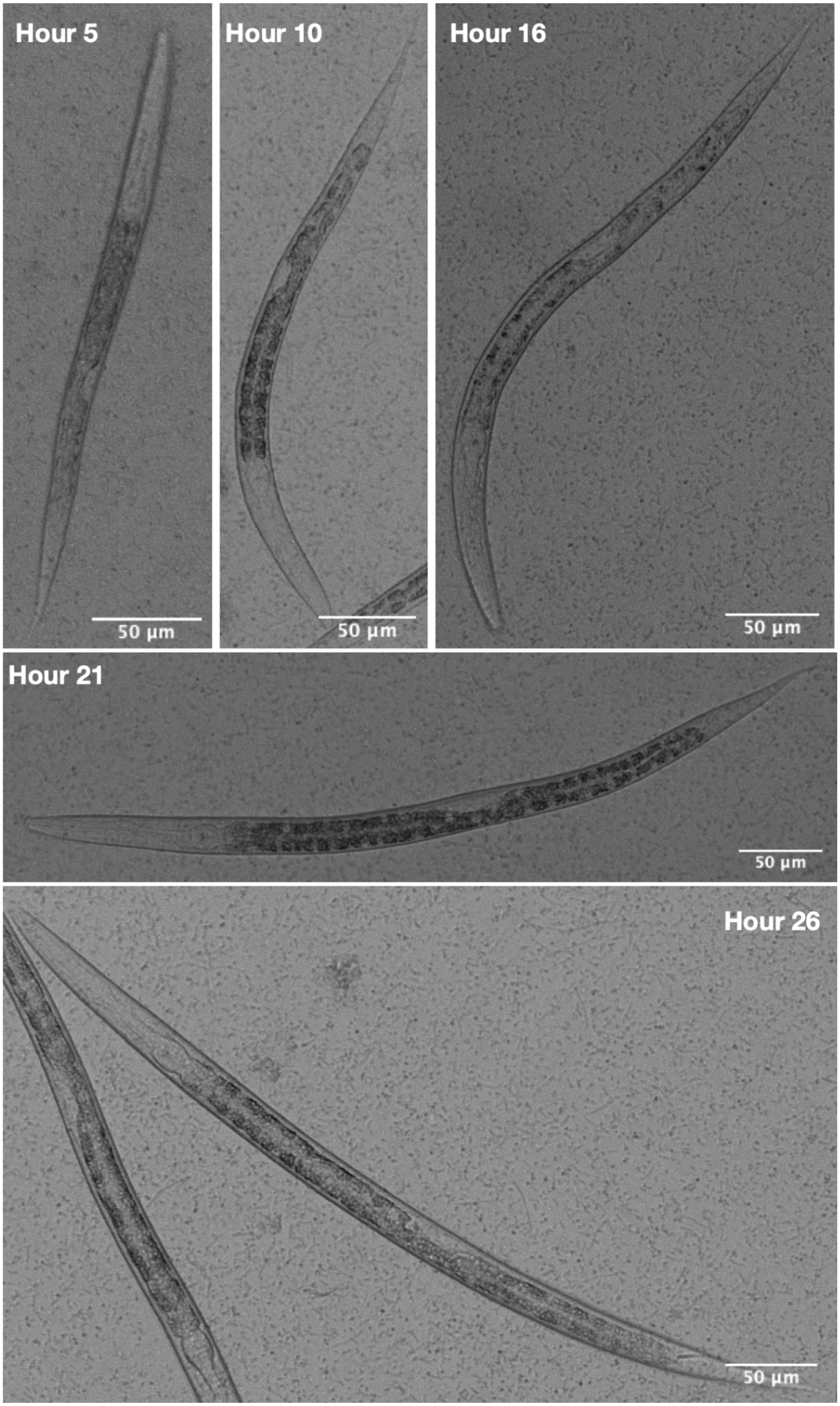
Hour 5
50 µm
Hour 10
50 µm
Hour 16
50 µm
Hour 21
50 µm
Hour 26
50 µm

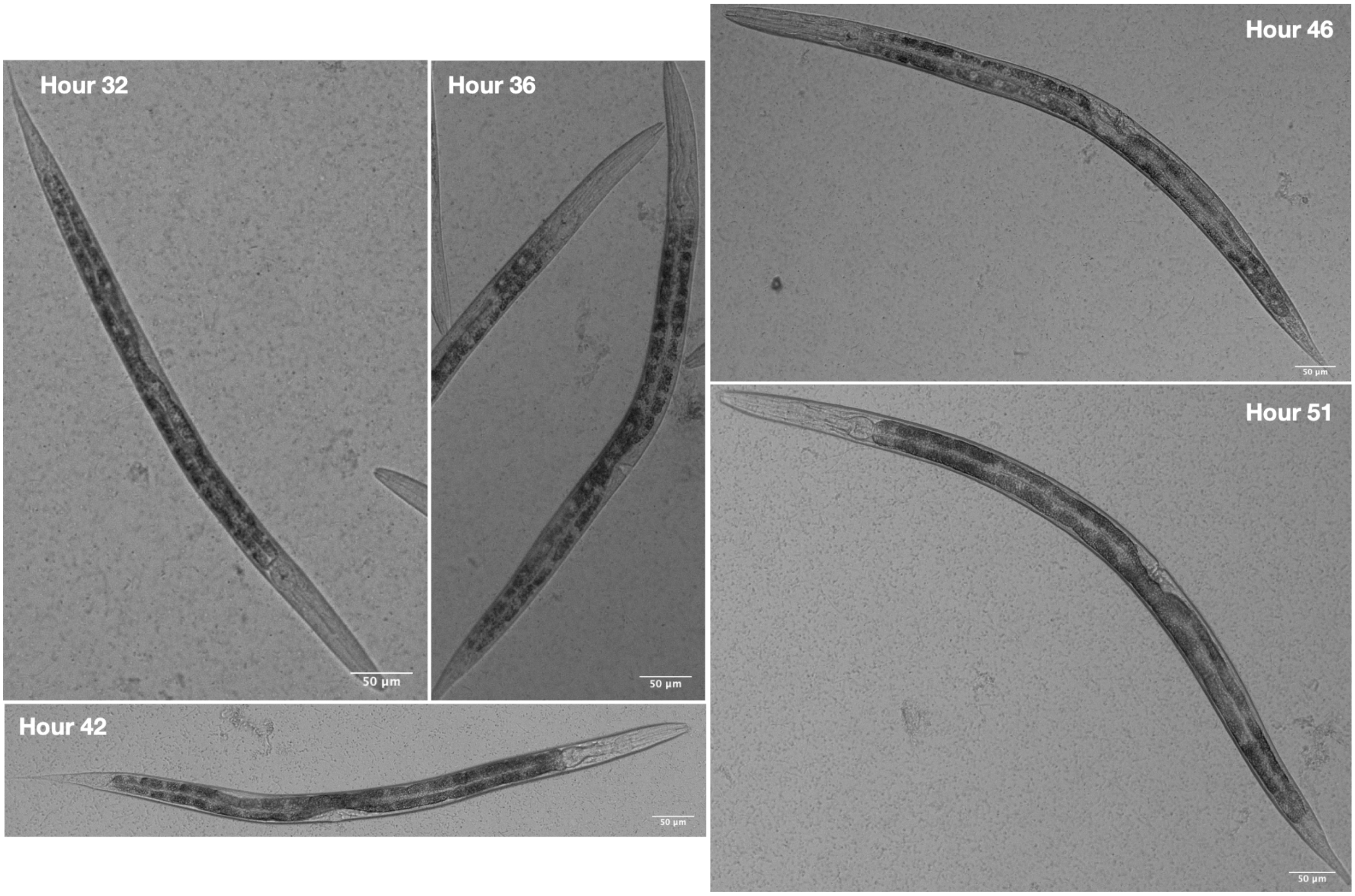

S2 Fig. Images of animals.
Images of animals taken over the 51 hour growth experiment.

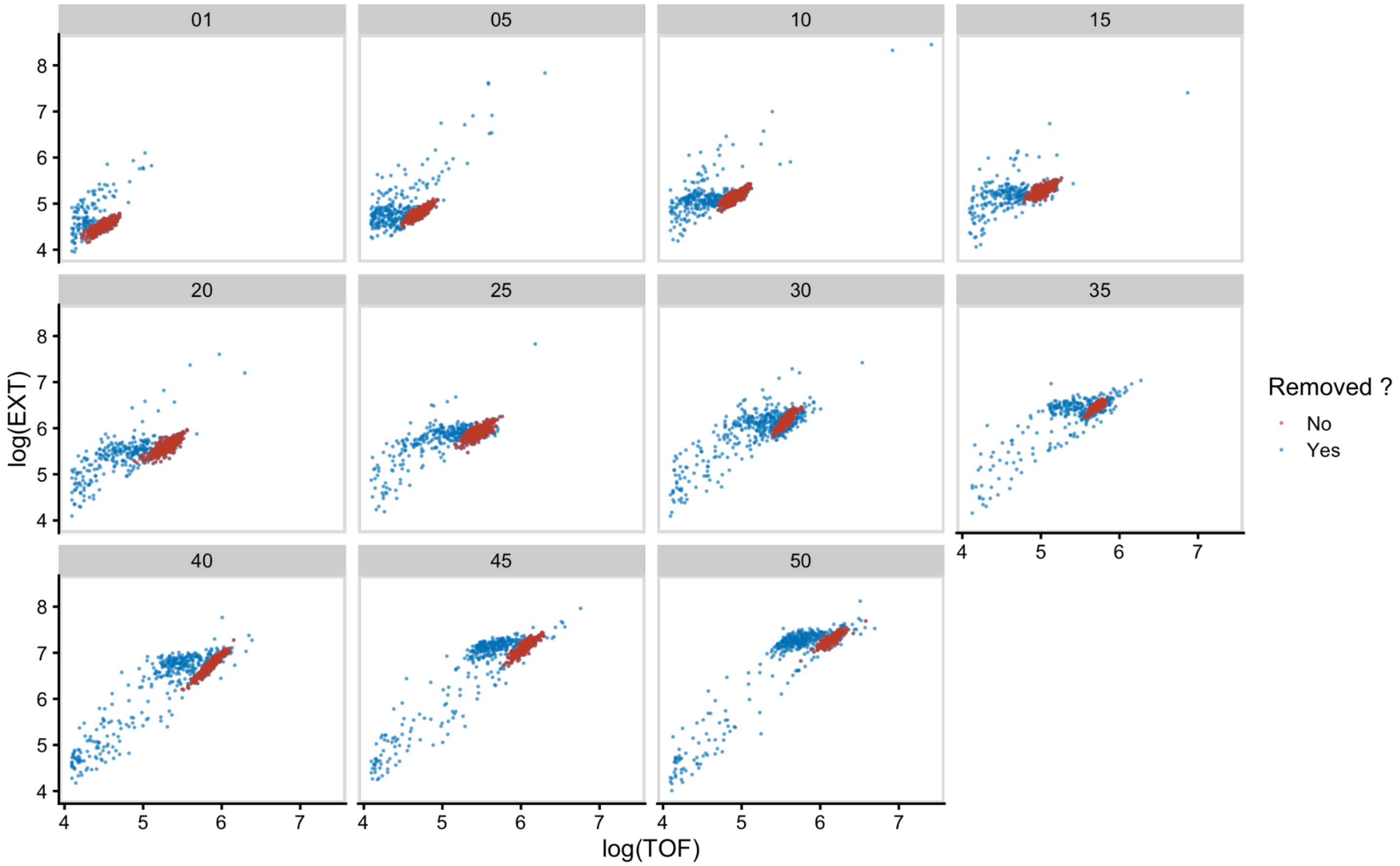

S3 Fig. Mixture modeling of COPAS BIOSORT data was used to prune data.
Mixture models of Gaussian distributions were fit to log transformed animal length (x-axis) and log transformed optical extinction (y-axis). Data from each hour of the experiment were separately analyzed and processed to remove clusters that did not include animal objects. Panels indicate experimental hours from which data were taken.

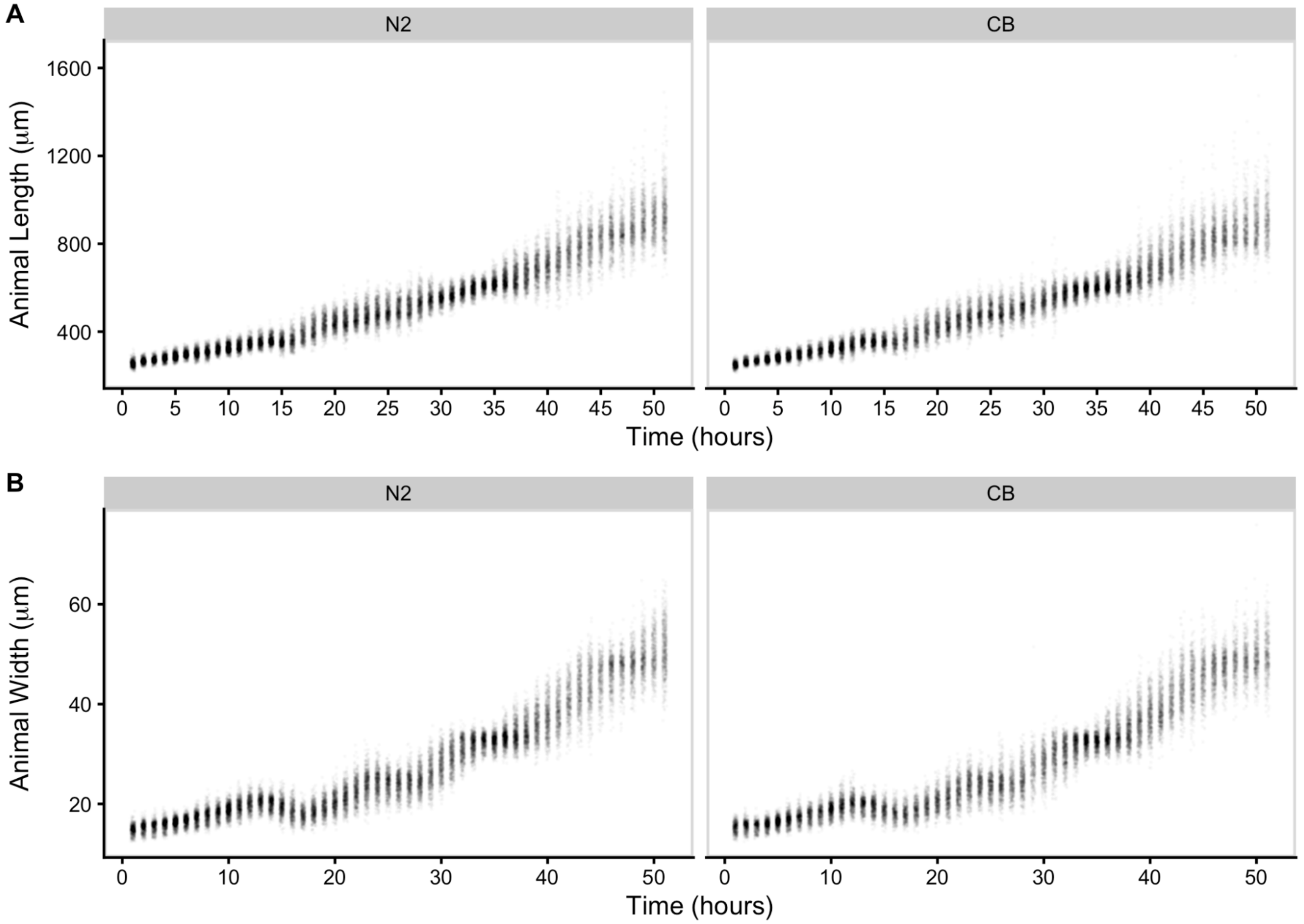

S4 Fig. Pruned measurements of animal size.
COPAS BIOSORT data of animal length (A) and width (B) after the removal of non-animal objects using model-based clustering methods. Each point represents an individual object that was measured.

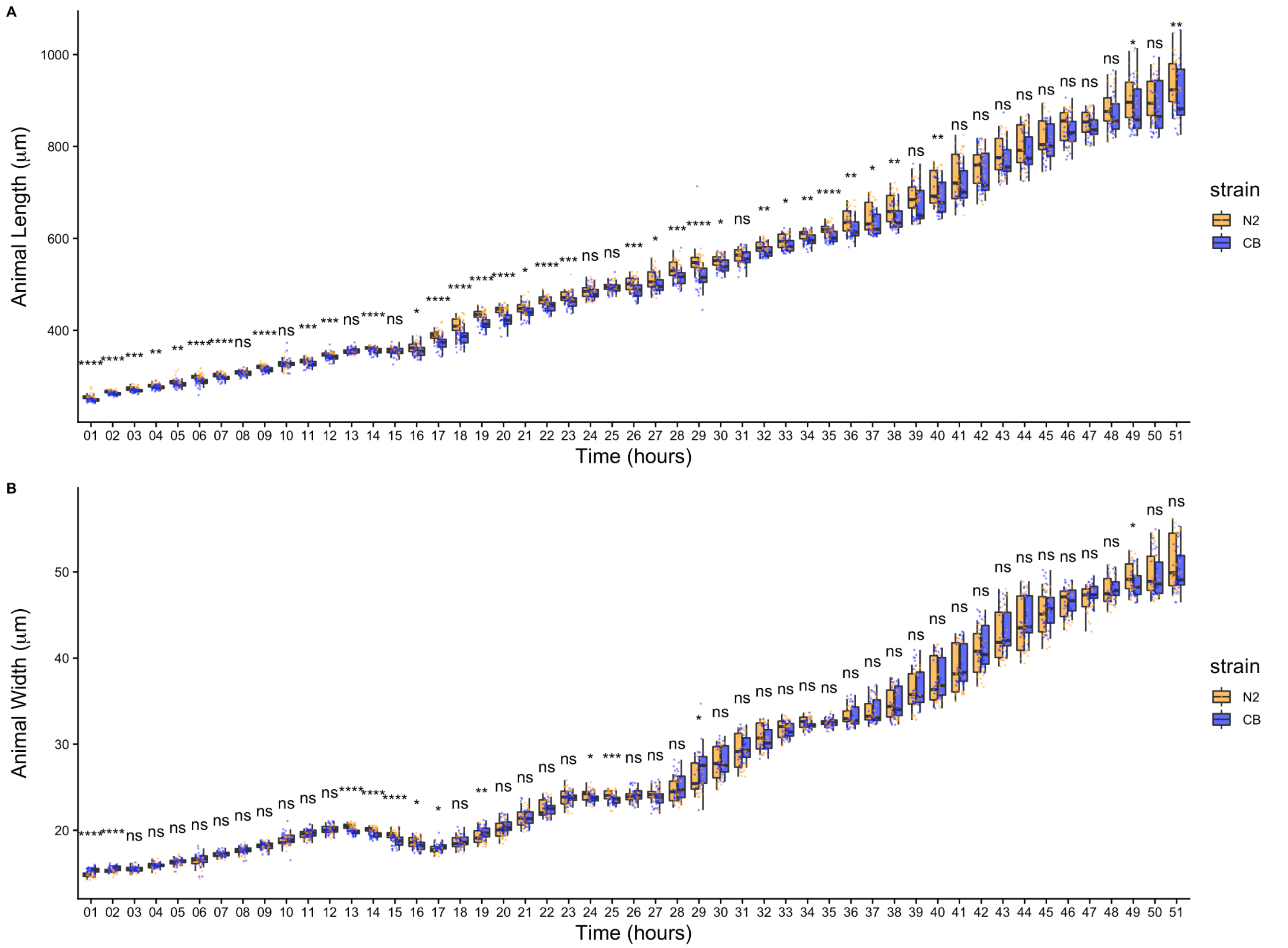

S5 Fig. Comparison of means across developmental time points.
Tukey boxplots of mean length **(A)** and mean width **(B)** for N2 (orange) and CB4856 (blue) over developmental time. The horizontal line in the middle of the box is the median, and the box denotes the 25th to 75th quantiles of the data. The vertical line represents the 1.5 interquartile range. Inset plots magnify mean animal size measurements from hour 48. Each point corresponds to the mean of a population of animals in each well. Statistical significance was calculated using a Wilcoxon test (ns = non-significant (p-value > 0.05); *, **, ***, and **** = significant (p-value < 0.05, 0.01, 0.001, or 0.0001, respectively).

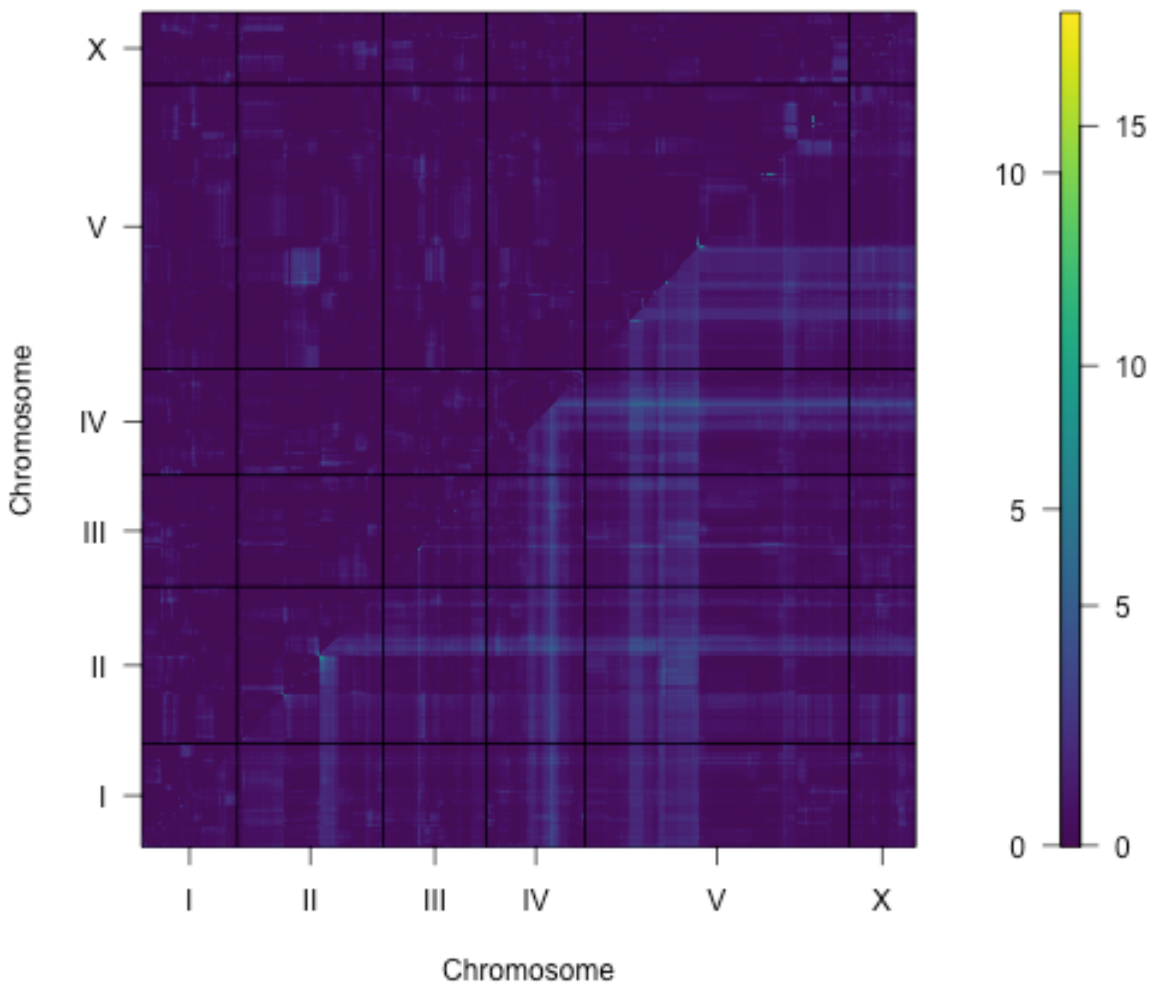

S6 Fig. Two-dimensional genome scan for mean animal length.
Log of the odds (LOD) scores are shown for each pairwise combination of loci, split by chromosome. The upper-left triangle contains the epistasis LOD scores and the lower-right triangle contains the LOD scores for the full model. LOD scores are colored, increasing from purple to green to yellow. The LOD scores for the epistasis model are shown on the left of the color scale and the LOD scores for the full model are shown on the right.

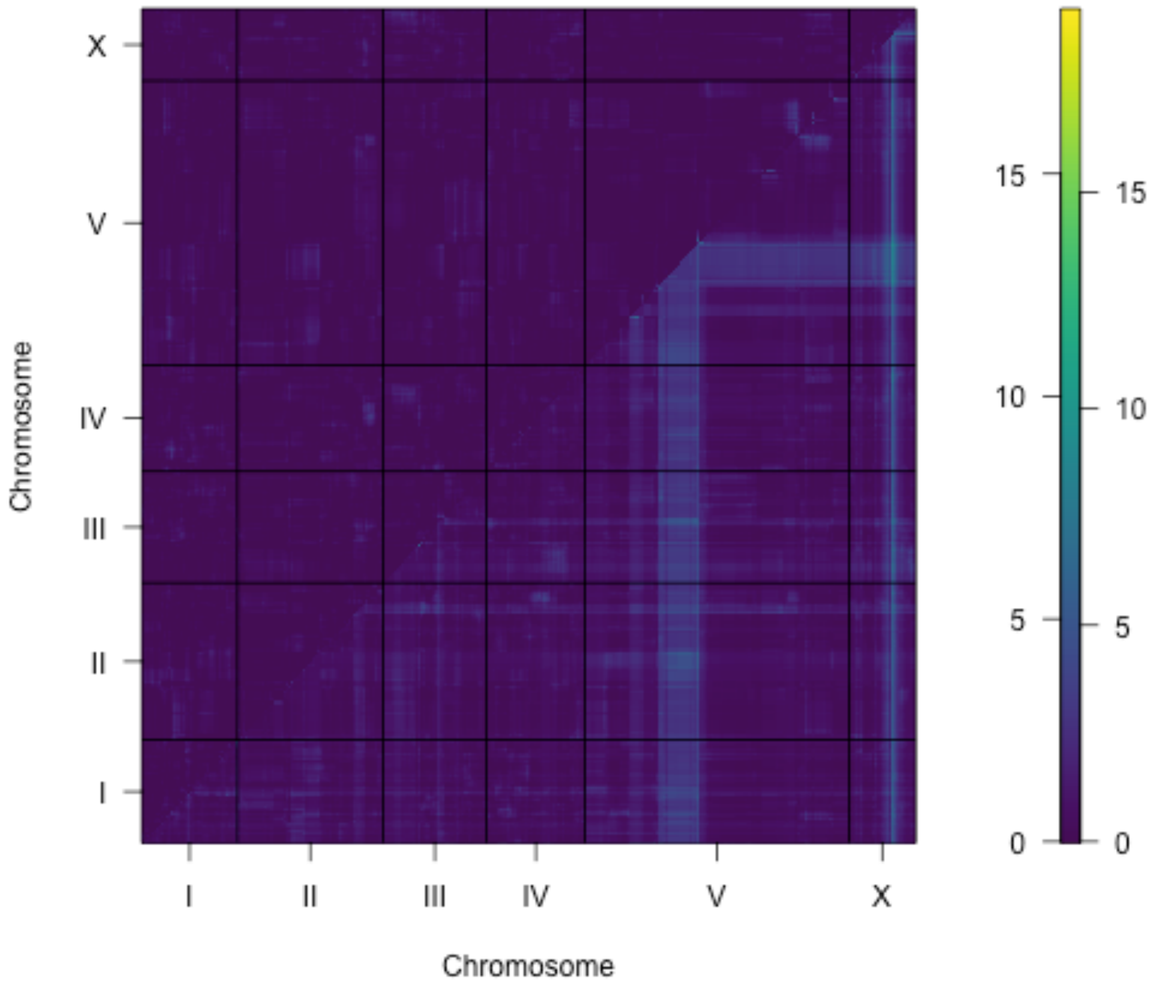

S7 Fig. Two-dimensional genome scan for mean animal width.
Log of the odds (LOD) scores are shown for each pairwise combination of loci, split by chromosome. The upper-left triangle contains the epistasis LOD scores and the lower-right triangle contains the LOD scores for the full model. LOD scores are colored, increasing from purple to green to yellow. The LOD scores for the epistasis model are shown on the left of the color scale and the LOD scores for the full model are shown on the right.

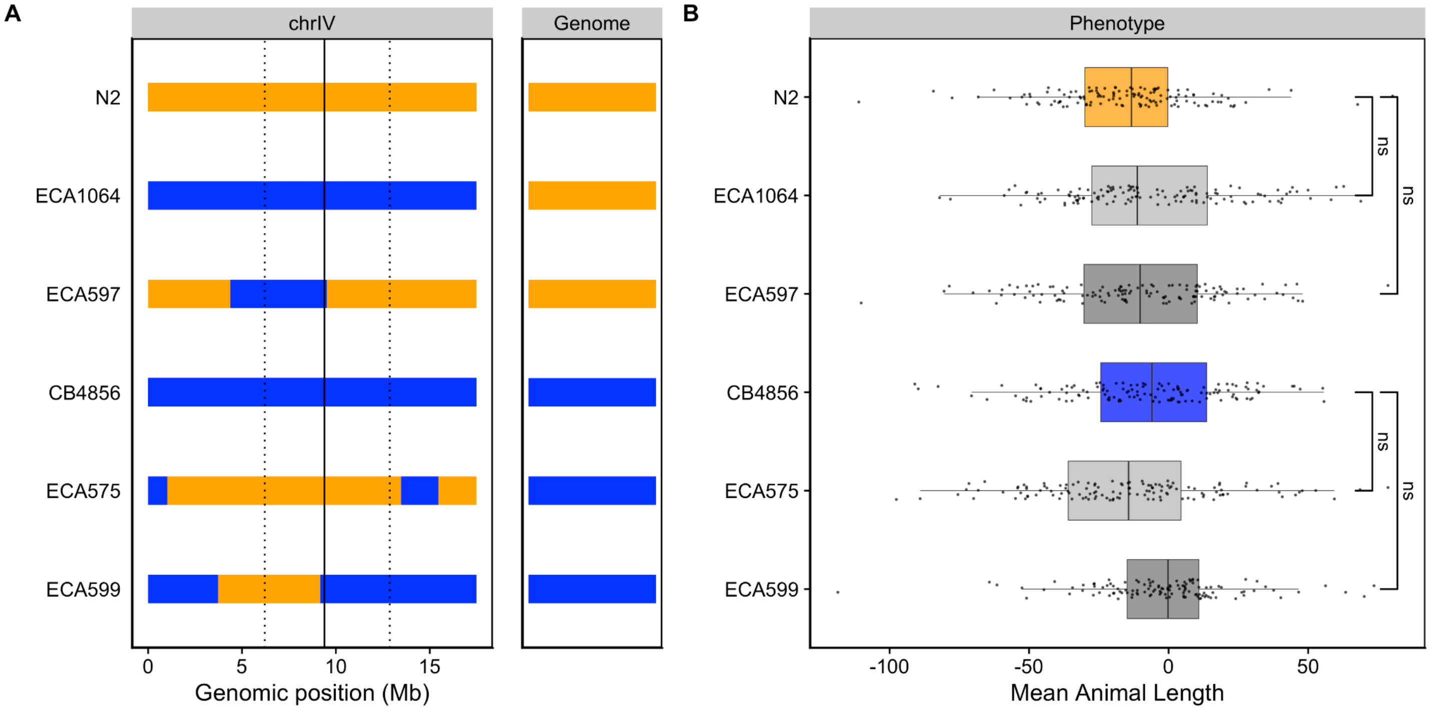

S8 Fig. Validating the chromosome IV length-associated QTL.
Strain genotypes are displayed as colored rectangles (N2: orange, CB4856: blue) for chromosome IV (left) and in general for the rest of the chromosomes (right). The solid vertical line represents the peak marker of the QTL. The dashed vertical lines represent the confidence interval. (B) Residual mean animal length (x-axis) is plotted as Tukey box plots against strain (y-axis). Each point corresponds to the mean length of a population of animals from each well in an assay. The boxes for the parental strains are colored: N2, orange; CB4856, blue. Statistical significance was calculated by Tukey's HSD (ns = non-significant, *p*-value > 0.05).

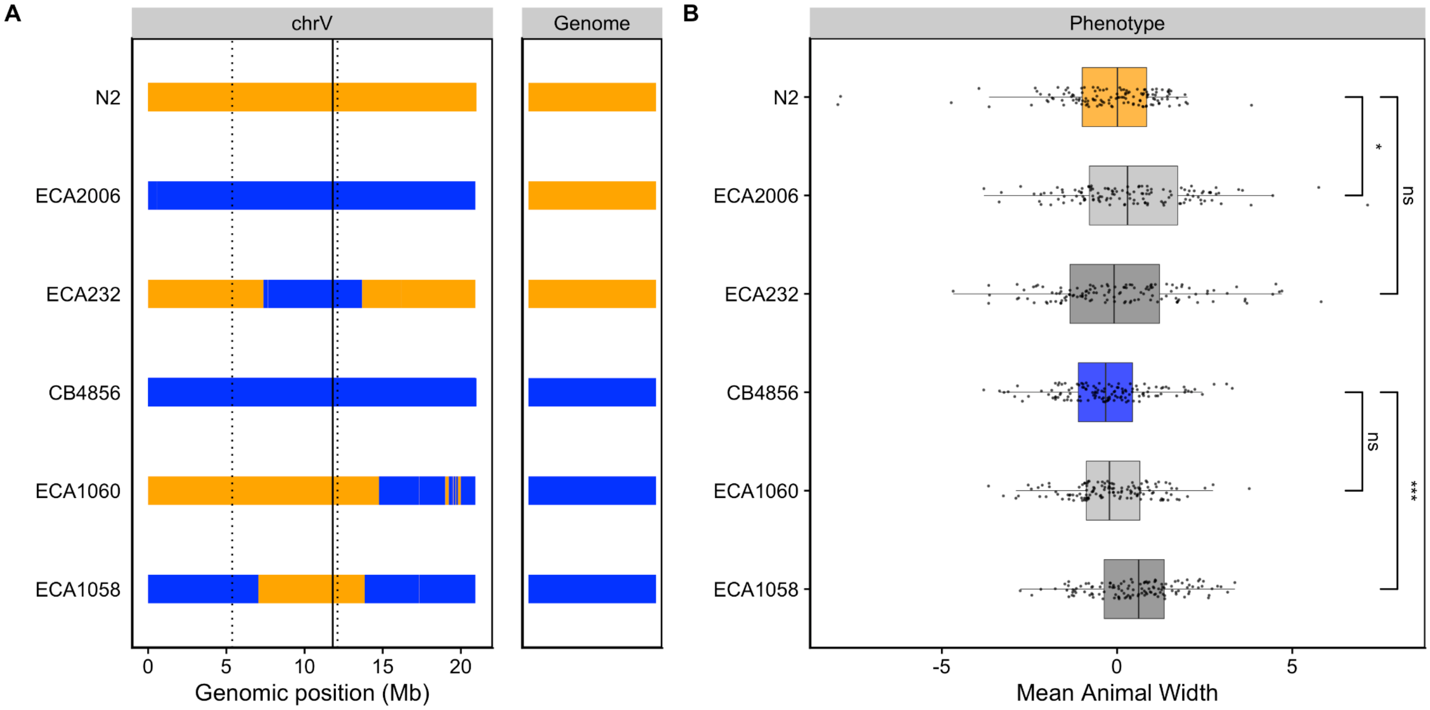

S9 Fig. Validating the chromosome V width-associated QTL.
Strain genotypes are displayed as colored rectangles (N2: orange, CB4856: blue) for chromosome V (left) and in general for the rest of the chromosomes (right). The solid vertical line represents the peak marker of the QTL. The dashed vertical lines represent the confidence interval. (B) Residual mean animal length (x-axis) is plotted as Tukey box plots against strain (y-axis). Each point corresponds to the mean width of a population of animals from each well in an assay. The parental strains are colored: N2, orange; CB4856, blue. Statistical significance was calculated by Tukey's HSD (ns = non-significant, *p*-value > 0.05; * and *** = significant, *p*-value < 0.05 or 0.001 respectively).

S1 Text. Reagents used to generate CSSs and NILs.

List of RIAILs used:

Set 2:

QX240, QX241, QX242, QX243, QX244, QX245, QX248, QX250, QX252, QX253, QX254, QX258, QX261, QX263, QX264, QX265, QX266, QX267, QX268, QX269, QX270, QX271, QX272, QX273, QX274, QX275, QX276, QX277, QX278, QX279, QX280, QX281, QX282, QX283, QX284, QX285, QX286, QX287, QX288, QX289, QX290, QX291, QX293, QX294, QX295, QX296, QX297, QX298, QX299, QX300, QX301, QX302, QX303, QX304, QX305, QX306, QX307, QX309, QX310, QX311, QX314, QX315, QX316, QX318, QX319, QX320, QX321, QX322, QX323, QX324, QX325, QX326, QX327, QX328, QX329, QX330, QX331, QX332, QX333, QX334, QX335, QX336, QX337, QX338, QX339, QX340, QX341, QX343, QX345, QX346, QX347, QX348, QX349, QX350, QX352, QX353, QX354, QX355, QX356, QX357, QX358, QX359, QX360, QX361, QX362, QX363, QX364, QX365, QX366, QX367, QX368, QX369, QX370, QX371, QX372, QX373, QX374, QX375, QX376, QX377, QX378, QX379, QX380, QX381, QX382, QX383, QX384, QX385, QX386, QX387, QX389, QX390, QX391, QX392, QX393, QX394, QX395, QX396, QX397, QX398, QX399, QX400, QX401, QX402, QX403, QX404, QX405, QX406, QX407, QX408, QX409, QX410, QX411, QX412, QX413, QX414, QX415, QX416, QX417, QX418, QX419, QX420, QX421, QX423, QX424, QX426, QX427, QX428, QX429, QX430, QX431, QX432, QX433, QX434, QX435, QX436, QX437, QX438, QX439, QX440, QX441, QX443, QX444, QX445, QX446, QX447, QX448, QX449, QX450, QX451, QX452, QX453, QX454, QX455, QX456, QX457, QX458, QX459, QX460, QX461, QX463, QX464, QX465, QX466, QX467, QX468, QX469, QX470, QX471, QX472, QX473, QX474, QX475, QX476, QX477, QX478, QX479, QX480, QX481, QX482, QX483, QX484, QX485, QX486, QX487, QX488, QX489, QX490, QX491, QX492, QX493, QX494, QX495, QX496, QX497, QX498, QX500, QX501, QX503, QX506, QX508, QX511, QX513, QX514, QX515, QX520, QX521, QX523, QX524, QX525, QX526, QX527, QX528, QX529, QX530, QX531, QX533, QX534, QX538, QX539, QX540, QX542, QX545, QX549, QX550, QX551, QX553, QX554, QX555, QX556, QX557, QX559, QX560, QX561, QX563, QX564, QX565, QX570, QX572, QX573, QX579, QX580, QX583, QX584, QX585, QX587, QX588, QX594, QX596, QX597, QX598

Reagents to generate CSSs and NILs:

Strain	Genotype	Constructed from	Left primer	Right primer
ECA232	*eanIR152*[V, CB4856>N2]	QX450xN2	oECA799 & oECA800	oECA745 & oECA746
ECA575	*eanIR324*[IV, N2>CB4856]	N2xCB4856	oECA1132 & oECA1133	oECA1135 & oECA1136
ECA597	*eanIR330*[IV, CB4856>N2]	ECA231xN2	oECA781 & oECA782	oECA857 & oECA858
ECA599	*eanIR332*[IV, N2>CB4856]	ECA598xCB4856	oECA781 & oECA782	oECA857 & oECA858
ECA828	*eanIR359*[X, N2>CB4856]	N2xCB4856	oECA1313 & oECA1314	oECA1246 & oECA1247
ECA929	*eanIR411*[X, CB4856>N2]	N2xCB4856	oECA1313 & oECA1314	oECA1246 & oECA1247
ECA1058	*eanIR433*[V, N2 > CB4856]	ECA1029xCB4856	oECA1408 & oECA1409	oECA1341 & oECA1342
ECA1060	*eanIR435*[V, N2 > CB4856]	ECA554xCB4856	oECA745 & oECA746	oECA763 & oECA764
ECA1064	*eanIR439*[IV, CB4856>N2]	N2xCB4856	oECA1131 & oECA1132	oECA1135 & oECA1136
ECA2006	*eanIR446*[V, CB4856>N2]	N2xCB4856	oECA1141 & oECA1142	oECA1147 & oECA1148

Primers:

Primer	Genomic position	Sequence
oECA745	V:13,110,045	tgcagaggtggagtaaccct
oECA746	V:13,110,045	ctcggtctctcccccactaa
oECA763	V:15,121,356	cgcacattctttatttctggcg
oECA764	V:15,121,356	atcggccgtttttcacctga
oECA781	IV:5,110,734	gagcactttggcgactttcg
oECA782	IV:5,110,734	tccgggcaaattagtgtggc
oECA799	V:7,862,556	ttctcgctactggaacacgc
oECA800	V:7,862,556	tcaagaagcgttgggaagtct
oECA1131	IV:1,039,851	tacccaccgcatcaaaacca
oECA1132	IV:1,039,851	acaggcgttcaaagacacca
oECA1135	IV:17,317,014	tttcagacaggaaagcgcct
oECA1136	IV:17,317,014	gttgagagatccggaccgac
oECA1141	V:144,547	ctcatgggagtaacctgggc
oECA1142	V:144,547	cggtgacaacggagaatcca
oECA1147	V:20,622,851	gtttagtaccagcggggcat
oECA1148	V:20,622,851	tgcattccgacccaagagac
oECA1246	X:11,696,902	tgcggtgggacttttcttgt
oECA1247	X:11,696,902	gtcccagcatgtaaccgtct
oECA1313	X:8,038,337	gctgtgcaggactggatgta
oECA1314	X:8,038,337	tgctttctgatctgtgccgt
oECA1341	V:7,104,674	cccatccccacaatgtttcg
oECA1342	V:7,104,674	aatcgacgagtggcacttgt
oECA1408	V:3,778,859	cacgtgcccttttgcaatga
oECA1409	V:3,778,859	gagctcccggaaaactcgaa
